# Presentation of a weight bias internalization tool for use in pregnancy and a call for future research: A commentary

**DOI:** 10.1016/j.obpill.2024.100107

**Published:** 2024-03-07

**Authors:** Taniya S. Nagpal, Nicole Pearce, Kristi B. Adamo

**Affiliations:** a Faculty of Kinesiology, Sport, and Recreation, University of Alberta, Edmonton, Canada; b Obesity Canada, Edmonton, Canada; c School of Kinesiology, University of Ottawa, Ottawa, Canada

**Keywords:** Pregnancy, Obesity, Weight stigma

## Abstract

**Background:**

Emerging evidence has shown that weight stigma is a concern during pregnancy, with several studies documenting common sources including healthcare, the media and interpersonal networks. Experiencing weight stigma may lead to weight bias internalization (WBI), whereby individuals accept and self-direct negative weight-related stereotypes, and limited research has assessed this in the context of pregnancy. Pregnancy is unique in terms of weight changes as many individuals will experience gestational weight gain (GWG). Accordingly, a WBI tool that accounts for GWG may be a more population-specific resource to use.

**Methods:**

This commentary presents a pregnancy-specific WBI tool that accounts for GWG. The validated Adult WBI scale was modified to include ‘pregnancy weight gain’. This commentary also presents a brief summary of research that has assessed WBI in pregnancy and recommendations for future work.

**Results:**

Recommended future work includes validation of the pregnancy-specific WBI tool and prospective examinations of weight stigma and WBI in pregnancy and implications on maternal and newborn outcomes.

**Conclusion:**

Ultimately this research may inform development of interventions and resources to mitigate weight stigma and WBI in pregnancy and overall may contribute to improving prenatal outcomes and experiences.

## 1 Background

Weight stigma, defined as negative misconceptions and stereotypes associated with weight, is a well-documented barrier to effective obesity prevention and management [1,2]. Weight stigma is initially a socially constructed phenomenon that facilitates systemic and social barriers for individuals at any age who have a larger body or obesity, and can lead to weight-based discrimination and mistreatment [1,2]. For example, several studies have documented the implications of weight stigma amongst school-aged children and youth who have obesity, including bullying, poor academic performance, lack of engagement in physical education classes, and increased risk of depression and anxiety [[3], [4], [5]]. For adults, weight stigma can occur in settings such as at work (e.g., lack of promotion due to body size and weight), healthcare (e.g., refusal of healthcare services, poor communication with healthcare providers), within interpersonal networks (e.g., weight-related teasing and negative commentary), and in public spaces (e.g., negative judgement in airplanes and grocery stores) [1,[6], [7], [8]]. Experiencing weight stigma in adulthood has been associated with mental health disorders, limited or poor communication with healthcare providers, delayed care, avoidance of physical activity, eating disorders, and physical health consequences such as elevated blood pressure [2,[9], [10], [11]]. Accordingly, weight stigma has been described as the ‘driver’ of obesity as it stands as a pervasive barrier to receiving adequate support for weight management and leads to devaluing individuals who have larger bodies, which further promotes comorbidities like mental health disorders [2,6]. A global call to action highlighted that a key direction forward to improve obesity care is to mitigate weight stigma across the life course [1].

Weight bias internalization (WBI) refers to when an individual accepts and self-directs weight stigmatizing discourse [12,13]. In fact, previous studies have suggested that WBI may catalyse the pathway from weight stigma to poor physical and mental health outcomes [2,14]. Experiencing WBI in adulthood has been associated with eating disorders, negative health related quality of life, and reduced self-efficacy for physical activity [12,13,15,16]. In addition, WBI directly harms psychosocial health, such as reducing one's self-esteem, thus leading to individuals who have larger bodies agreeing with societal perspectives that their weight and obesity is a personal failure [12]. Females and women reportedly are more likely to internalize weight biases than males and men, although all populations are subjected to weight stigmatizing messages that predominantly favour smaller bodies irrespective of sex and gender [13,17]. An emerging body of literature has explored weight stigma experienced during pregnancy, a time when females are often undergoing physical changes, including weight gain [18,19].

## 2 Weight stigma and weight bias internalization in pregnancy

Weight stigma during pregnancy is described as unique or distinct from weight stigma experienced outside of prenatal time frames, as weight gain and change is both clinically and socially expected [18]. Clinically, weight is monitored throughout gestation during prenatal appointments as a measure of pregnancy progression, and maternal and fetal health [18]. Socially, pregnancy is characterized as an ‘acceptable/expected’ time of weight gain. However, there are still persistent body ideals even during pregnancy [18]. Ideal pregnant bodies are described as small, with only a pronounced abdomen, resulting in weight stigmatization of individuals who have larger bodies [18]. In fact, studies that have examined the prevalence of weight stigma during pregnancy have consistently reported a positive correlation with maternal body mass index and obesity [[20], [21], [22]]. Obesity during pregnancy is associated with adverse perinatal outcomes such as increased risk of gestational diabetes, preeclampsia, and postpartum depression [23,24]. Accordingly, females who have obesity are often recommended to lose weight prior to conception to reduce the risk for negative health outcomes [25]. However, preconception weight loss expectations may inadvertently also contribute to weight stigma in pregnancy as those who do continue to have obesity during their pregnancy are subject to assumptions that they ‘failed’ to lose weight prior to conceiving and are consequently inflicting harm to themselves, the fetus, and the healthcare system [25,26]. For example, media analyses on the representation of obesity during pregnancy have highlighted the use of alarming or risk-mongering language when describing mothers who have larger bodies, and stigmatizing perceptions such as pregnant individuals with larger bodies are overeating or are not being physically active and therefore harming fetal development [26,27]. Weight stigmatizing discourse is also prevalent in the postpartum stage where there is pressure to lose weight rapidly [22]. Weight stigma experienced before, during and after pregnancy has been associated with negative outcomes inclusive of depression, gestational diabetes, and excessive gestational weight gain [20,22,28]. Documented sources of weight stigma in pregnancy include healthcare, the media, and interpersonal networks [21,27,29]. Moreover, there appears to be an intersectional relationship between weight stigma and minority racial and ethnic groups, where experiences may be nuanced based on cultural expectations surrounding ideal pregnant bodies and negative physical and mental health consequences are exacerbated [19]. For example, a large cross-sectional study including 501 pregnant and postpartum participants from the United States found that individuals who were Black or Hispanic were more likely to report engaging in emotional eating behaviours in response to weight stigma than White identifying groups [20]. Given that detrimental prenatal experiences can have negative health consequences for both the mother and future child, mitigating weight stigma during this time is of utmost importance [19].

Limited research has specifically sought to examine the implications of WBI during pregnancy; this is problematic given the evidence supporting internalization as a key mechanism that drives obesity related complications and comorbidities [13]. One cross-sectional study assessed the implications of WBI on gestational weight gain, postpartum weight retention and depression among 251 women residing in the United States at 6 and 12 months postpartum [30]. Findings showed that WBI was positively correlated with depression and postpartum weight retention [30]. Furthermore, WBI mediated the relationship between postpartum weight retention and depression [30]. Another cross-sectional study explored the relationship between prenatal WBI and breastfeeding intentions, continuation, and exclusivity at one month postpartum [31]. The participants included 103 women who had an overweight or obese body mass index and were residing in the United States, and no relationship was reported between WBI and the selected breastfeeding outcomes [31]. A limitation noted in both these studies was the use of the modified Adult Weight Bias Internalization Scale [32], which is not pregnancy-specific [30,31].

The Adult Weight Bias Internalization Scale is the most commonly used validated tool to assess WBI [32]. The scale consists of 10 statements and asks respondents to indicate their level of agreement on a 7-point likert scale [32]. A mean score is calculated, with a higher score indicating greater WBI [32]. This tool has consistently shown high reliability, and strong correlations with several biopsychological and behavioural outcomes including exercise avoidance, body image disturbances, depression, and weight gain [[12], [13], [14]]. As discussed above, pregnancy is distinct because of gestational weight gain and physical changes to body shape and size [18]. Therefore, it may be more accurate to measure WBI in pregnancy considering the physical changes in weight a person may be experiencing. To our knowledge, only one cross-sectional study administered in Canada and the United States has modified the Adult Weight Bias Internalization Scale to be pregnancy specific and inquire about internalization due to gestational weight gain [33].

Nagpal et al. (2021) in collaboration with Obesity Canada established an expert panel of researchers in gestational weight gain and weight bias, maternal healthcare providers, and individuals who have had lived experience of obesity in pregnancy [34]. The role of the panel was to modify the Adult Weight Bias Internalization Scale to specifically inquire about internalization as a result of pregnancy weight gain [34]. In the original scale, where it only indicated ‘weight’, the pregnancy version specified ‘weight gain during pregnancy’ [34]. Table 1 presents the pregnancy specific WBI scale. In a cross-sectional study, the scale was completed by a large sample of 336 pregnant individuals residing in Canada and the United States [33]. Findings indicated that WBI was higher among individuals who had been diagnosed with obesity in pregnancy, had a body mass index ≥30.0 kg/m^2^, and had gained above Institute of Medicine gestational weight gain recommendations [33]. Using the pregnancy-specific WBI scale may be more appropriate in pregnancy as it would consider the physical weight-related changes being experienced during this time. However, as mentioned, there is a paucity of research that has assessed WBI in pregnancy in general and below we have highlighted important directions forward that would aid in addressing this gap.

**Table 1:** Pregnancy weight gain specific weight bias internalization scale items(33).

1. I am less attractive than other people who are pregnant because of my weight gain during pregnancy.
2. I feel anxious about my weight gain during pregnancy because of what other people might think of me.
3. I wish I could drastically change my weight gain during pregnancy.
4. Whenever I think about my weight gain during pregnancy, I feel depressed.
5. I am disappointed in myself because of my weight gain during pregnancy.
6. My weight gain is a major way that I judge my value as a pregnant person.
7. I do not feel I deserve to have a fulfilling pregnancy experience because of my weight gain.
8. I am comfortable with my pregnancy weight gain.∗
9. I don't feel like my true self because of my weight gain during pregnancy.
10. I do not understand how anyone would want to socialize with me because of my weight gain during pregnancy.

a. All items are assessed on a 7-point scale with a higher score indicating greater agreement with the statement. A higher mean score reflects greater weight bias internalization.

b. The original scale includes 11-items, however item 1 has consistently demonstrated poor internal consistency in previous studies thus was removed (Pregnancy modification of the item: As a person with a larger body in pregnancy, I feel that I am just as competent as anyone).

c.∗Indicates reverse scoring.

## 3 Future directions for weight bias internalization in pregnancy research

Critical future directions for WBI in pregnancy research includes validation of the population-specific measurement tool and prospective examinations with maternal and newborn outcomes, ultimately leading to development and testing of interventions to reduce both weight stigma and WBI in pregnancy. Although the previous study that administered the pregnancy weight gain specific WBI tool demonstrated high internal consistency [33], validity of the scale remains to be established. In particular, following similar procedures as the validation of the modified Adult Weight Bias Internalization Scale may be a necessary next step [32]; this can include measuring its construct validity with other related measures such as body image in pregnancy, and the predictive value of psychological consequences that have a well-established relationship with WBI (e.g., disorder eating, reduced self-esteem, or depressive symptoms). These processes would also contribute to further advancing our knowledge on the relationship between WBI specifically in pregnancy and health and behavioural outcomes which remains a glaring gap. Further research should consider variation in experiences of and responses to weight stigma amongst diverse pregnant populations, such as the potentially unique experiences of individuals who identify with minority racial and ethnic groups, which may have cultural expectations that facilitate or protect against weight stigmatizing discourse.

In addition to cross-sectional studies, it would be prudent to assess prospectively how WBI may change as pregnancy progresses and the potentially evolving relationship with physical and psychological outcomes. As gestational weight gain is progressive, WBI may vary across trimesters and be related to changing health outcomes such as the development of any prenatal complications (e.g., gestational diabetes), and gestational weight gain itself. For example, an individual who may have been meeting Institute of Medicine gestational weight gain guidelines in the second trimester but experienced excessive weight gain in the third trimester may also have changes in their WBI, and this relationship could be evaluated in prospective longitudinal studies across pregnancy. In addition to weight gain, pregnancy can bring several other physical changes, such as shortness of breath, potential pain in the lower back, sleep disturbances, and water retention. Future research may also need to expand the existing tool that considers primarily only psychological constructs, to account for physical bodily changes that occur throughout pregnancy that may still be related to weight changes and thus influence or be influenced by WBI. Additionally, the original Adult Weight Bias Internalization Scale [32] has been used in the postpartum [31], however it may be plausible that a postpartum-specific tool that considers factors like internalized pressure to return to pre-pregnancy weight needs to be developed. The usefulness of this tool, or the already validated adult scale in the postpartum is not well established and requires further investigation.

**Fig. 1:**
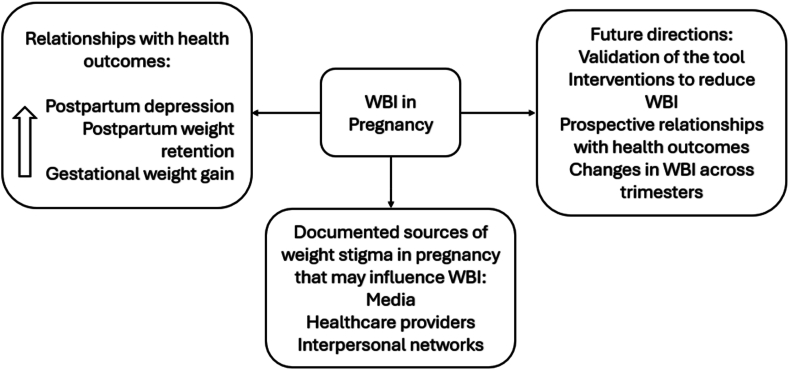
Summary of research and future directions on weight bias internalization during pregnancy. WBI - Weight bias internalization

Overall, there is limited research on WBI (summarized in Fig. 1) in pregnancy and its potential implications on outcomes. By having a validated assessment tool and high-quality studies that assess the relationship with maternal and newborn outcomes, the evidence-base can support and strengthen development of interventions to address WBI in pregnancy. Arguably, it is already established that weight stigma does indeed occur during pregnancy and individuals with obesity are particularly vulnerable [19]. However, most studies to date have focused on conceptualizing weight stigma in pregnancy, estimating prevalence, and highlighting relationships between the occurrence of weight stigma and health outcomes [19]. Limited work has assessed WBI, and interventions to address this at an individual-level have not been developed. Accordingly, this is a critical direction forward for research and clinical practice to improve prenatal care and outcomes. Importantly, given that weight stigma may occur before and after pregnancy as well, interventions should account for the potential nuances experienced in the preconception and postpartum period, which may include pressure to rapidly lose weight in an effort to conceive (preconception) or return to pre-pregnancy weight (postpartum) [18].

## 4 Conclusion

A critical gap in weight stigma and pregnancy research is our understanding of WBI during this unique time where most individuals experience gestational weight gain, and the relationship with health and behavioural outcomes. To measure WBI in pregnancy, it may be necessary to ensure that tools account for gestational weight-related changes, which may be possible with modifications made to the modified Adult Weight Bias Internalization Scale as described in Table 1. Critical directions forward include validation of the pregnancy-specific WBI measurement tool, a task that is presently underway in collaboration with Obesity Canada. It is also important to conduct prospective longitudinal studies that can measure the changes in WBI as pregnancy progresses and its relationships with perinatal outcomes. As the evidence base on weight stigma research in pregnancy continues to grow, inquiring about WBI will further strengthen initiatives for developing and testing interventions to support pregnant individuals and reduce weight stigma, which overall could result in improved delivery of prenatal care and pregnancy experiences.

### 4.1 Clinical takeaways

1. Weight stigma does occur during pregnancy from a variety of sources (e.g., communication with healthcare professionals, media representation of ideal pregnant bodies, comments from interpersonal networks) and this may result in pregnant individuals internalizing these stigmatizing values and views which consequently can impact physical and mental health.
2. Prenatal healthcare professionals can mitigate weight stigma in clinical settings by practicing sensitivity when discussing gestational weight gain, recognizing that their patient may experience weight stigma in pregnancy (e.g., feeling pressure to meet pregnancy body ideals).
3. Notably, the research on WBI in pregnancy is limited, including in clinical settings. Future work includes validating the proposed measurement tool, prospective examinations of relationships with physical and mental health outcomes, developing and testing interventions to reduce weight stigma and WBI, and assessing changes across trimesters; all of which could facilitate integration of WBI measurement and care within clinical practice.

## Authorship statement

TSN led the development of the pregnancy weight bias internalization scale and acquired funding. TSN, NP and KBA conceptualized this commentary. TSN drafted the manuscript with feedback from NP and KBA. All authors approved the final submission.

## Ethical review

As this is a commentary, ethical review was not required.

## Funding

The 10.13039/100021638Social Sciences and Humanities Research Council – Partnership Engage Grant (Award no. 892-2020-2040) with 10.13039/501100014344Obesity Canada.

## Declaration of artificial intelligence (AI) and AI-assisted technologies in the writing process

During the preparation of this work the authors did not use AI-assisted technologies.

## Declaration of competing interest

The authors declare no conflicts of interest. Nicole Pearce is an employee of Obesity Canada. Dr. Taniya Nagpal and Dr. Kristi Adamo are members of Obesity Canada.
